# Œsophagotomy for the Removal of an Artificial Denture

**Published:** 1886-12

**Authors:** 


### Editorial, Etc.

Œsophagotomy for the Removal of an Artificial Denture.—The following operation was performed before the students of the Medical and Dental Departments of the University of Maryland, by L. McLane Tiffany, M. D., Professor of Surgery.

On Sunday, November 14th, 1886, George K., (white), aged 32 years, residing in Baltimore county, Md , while at dinner, had the misfortune to partially swallow his artificial teeth, consisting of a rather narrow vulcanite plate for the upper jaw, to which were attached three incisor teeth, one lateral incisor having been lost from the plate. The denture was arrested in its passage downwards, producing intense pain and partially obstructing respiration, while deglutition, even of liquids, was rendered impossible. A physician was summoned, who detected the plate in the upper portion of the œsophagus, but all efforts to remove it or force it into the stomach were futile. Sufficient opium to relieve the pain having been administered, on the following day, Monday, he was brought by his physician to the Infirmary of the University of Maryland, and placed under the care of Dr. L. McLane Tiffany, Professor of Surgery. On the same afternoon, the patient having been etherized, careful attempts to remove the plate were made, but it was so firmly impacted in the upper portion of the œsophagus that all efforts for its removal failed. On Tuesday, in the presence of the medical and dental classes, the patient was again etherized and efforts made to remove the plate through the mouth, but without success.

The patient lying on his back with his face turned to the right, so as to render the tissues of the left side of the neck tense, Prof. Tiffany made an incision about four inches in length through the integument over the depression between the trachea and the sterno-mastoid muscle. The anterior jugular vein was cut and ligated, and the incision extended from opposite the upper border of the thyroid cartilage nearly as low as the sterno clavicular articulation. The platysma myoides muscle, and the cervical fascia were then divided. The edges of the wound being held apart with retractors, the omo hyoid muscle was drawn outwards, and the sterno-hyoid and sterno-thyroid muscles inwards. The carotid sheath, with the contained vessels, was exposed and carefully drawn outwards, while the thyroid gland was separated as far as necessary and drawn inwards. The larynx and trachea were drawn somewhat forwards, and the finger passed behind where the foreign body could be distinctly felt through the œsophageal wall.

An incision large enough to admit the finger, was then made into the œsophagus, care being taken to avoid the recurrent laryngeal nerve, through which the exact position of the set of teeth was ascertained. Forceps were then introduced and the plate removed intact. The wound after being thoroughly cleansed, was dressed with antiseptic gauze and absorbent cotton, no sutures being employed. On the following Thursday, the patient was walking about his room with a normal temperature and pulse. He was fed by means of a stomach-tube for six days, after which he was able to swallow liquid food with little or no pain, and the external wound had nearly closed.

